# ﻿Identification and fungicide sensitivity of *Microdochiumchrysopogonis* (Ascomycota, Amphisphaeriaceae), a new species causing tar spot of *Chrysopogonzizanioides* in southern China

**DOI:** 10.3897/mycokeys.100.112128

**Published:** 2023-12-06

**Authors:** Xiang Lu, Mengxian Mai, Wenhui Tan, Muyan Zhang, Jie Xie, Yi Lu, Xue Li Niu, Wu Zhang

**Affiliations:** 1 School of Life Sciences and Technology, Lingnan Normal University, Zhanjiang 524048, China Lingnan Normal University Zhanjiang China; 2 School of Geographical Science, Lingnan Normal University, Zhanjiang 524048, China Lingnan Normal University Zhanjiang China

**Keywords:** fungicide sensitivity, multilocus phylogeny, new taxon, pathogenicity, tar spot

## Abstract

Vetiver grass (*Chrysopogonzizanioides*) has received extensive attention in recent years due to its diverse applications in soil and water conservation, heavy metal remediation, as well as essential oil and phenolic acids extraction. In 2019, the emergence of tar spot disease on *C.zizanioides* was documented in Zhanjiang, Guangdong Province, China. Initially, the disease manifested as black ascomata embedded within leaf tissue, either scattered or clustered on leaf surfaces. Subsequently, these ascomata became surrounded by fisheye lesions, characterised by brown, elliptical, necrotic haloes, which eventually coalesced, resulting in leaf withering. Koch’s postulates demonstrated that the fungus isolated from these lesions was the causal agent. Microscopic examination showed that the pathogen morphologically belonged to *Microdochium*. The phylogenetic tree inferred from the combined ITS, LSU, *tub2* and *rpb2* sequences revealed the three isolates including GDMCC 3.683, LNU-196 and LNU-197 to be a novel species of *Microdochium*. Combining the results of phylogenetic, pathogenicity and morphological analyses, we propose a new species named *M.chrysopogonis* as the causal agent of *C.zizanioides* in southern China. The optimum growth temperature for *M.chrysopogonis* was determined to be 30 °C. The in vitro fungicide sensitivity of *M.chrysopogonis* was determined using a mycelial growth assay. Four demethylation-inhibiting (DMI) fungicides, including difenoconazole, flusilazole, propiconazole and tebuconazole and one methyl benzimidazole carbamate (MBC) fungicide, carbendazim, were effective against *M.chrysopogonis*, with mean 50% effective concentration (EC_50_) values of 0.077, 0.011, 0.004, 0.024 and 0.007 μg/ml, respectively. These findings provide essential references for the precise diagnosis and effective management of *M.chrysopogonis*.

## ﻿Introduction

Vetiver (*Chrysopogonzizanioides*) is one of the main grasses in tropical and subtropical areas (Maurya et al. 2023). With a large root system that penetrates deep into the soil, *C.zizanioides* is strongly tolerant to adverse environments, such as drought, salinity and heavy metals. Recently, vetiver has been widely utilised in various applications, such as land restoration, soil and water conservation and phytoremediation of heavy metal-contaminated soils (Chen et al. 2021). Moreover, the essential oil and phenolic acids extracted from vetiver root possess significant aromatic and biological properties, giving it an important role in perfumery, the food industry and medicine (David et al. 2019; Moon et al. 2020).

To our knowledge, four diseases on *C.zizanioides* have been reported, namely, leaf blight caused by *Curvulariatrifolii* in India (Babu et al. 2021), root and basal stem rot caused by *Gaeumannomycesgraminicola* (Lin et al. 2019), leaf spot caused by *Phomaherbarum* (Zhang et al. 2017) and leaf streak caused by *Stenocarpellachrysopogonis* (Jia et al. 2023).

*Microdochium* species were originally introduced with the type species, *M.phragmitis*, identified on the leaves of *Phragmitesaustralis* in Germany (Sydow 1924). Presently, there are 49 species included in this genus. However, only a subset of these species can induce diseases, primarily affecting grasses and cereals. For example, *M.albescens* (also referred to as *Monographellaalbescens*) typically induces leaf scald and grain discoloration in rice, leading to a global reduction in rice yield (Araujo et al. 2016; Dirchwolf et al. 2023). *M.bolleyi* is recognised for inducing root necrosis and basal rot in creeping bent grass in Korea, as well as causing root rot on triticale in Kazakhstan (Hong et al. 2008; Alkan et al. 2021). *M.nivale* and *M.majus* frequently result in the occurrence of pink snow mould or Fusarium Patch on wheat, barley and turf grass in cold to temperate regions (Ren et al. 2015; Abdelhalim et al. 2020). *M.opuntiae* leads to brown spotting on *Opuntia* (Braun 1995). *M.poae* triggers leaf blight disease in turf-grasses, such as *Poapratensis* and *Agrostisstolonifera* (Liang et al. 2019). *M.paspali* is recognised for its ability to cause leaf blight in seashore paspalum (*Paspalumvaginatum*) (Zhang et al. 2015). *M.panattonianum* has the potential to induce anthracnose in lettuce (Galea et al. 1986). *M.sorghi* is accountable for the formation of zonate leaf spots and decay on sorghum species (Stewart et al. 2019).

The application of fungicides has always been an effective approach for disease control. In recent decades, demethylation-inhibiting (DMI) fungicides have emerged as a significant and extensive group of fungicides, exhibiting notable efficacy in the control of diseases caused by the *Microdochium* genus. Notably, compounds such as prochloraz, difenoconazole, propiconazole, metconazole, myclobutanil, tebuconazole and triticonazole have shown substantial antifungal efficacy against *M.panattonianum*, *M.majus* and *M.nivale* (Wicks et al. 1994; Debieu et al. 2000; Glynn et al. 2008; Mao et al. 2023). Additionally, fungicide subgroups, including phenylpyrrole (PP) fungicides, such as fludioxonil, dicarboximides, such as iprodione and quinone outside inhibitors (QoIs), such as trifloxystrobin, have demonstrated noteworthy efficacy in the management of diseases induced by *M.nivale* (Glynn et al. 2008; Koch et al. 2015; Aamlid et al. 2017). Therefore, to promote effective control against tar spot of *C.zizanioides*, it is necessary to determine the sensitivity of the pathogen to fungicides.

The main objectives of this study were to identify the pathogenic fungi causing tar spot of *C.zizanioides* in southern China on the basis of morphological characteristics and multigene sequence analysis; to determine the pathogenicity to *C.zizanioides*; and to determine the inhibitory effect of fungicides against mycelial growth of the pathogen.

## ﻿Materials and methods

### ﻿Sample collection and fungal isolation

Leaves exhibiting symptoms of tar spot on *C.zizanioides* were collected in fields of the Grass Research Station of Lingnan Normal University (LNU), Zhanjiang, Guangdong, China. Leaf segments (0.5 × 0.5 cm) from the transition zone from diseased to healthy tissue were cut and surface-sterilised for 30 s with 75% ethanol and 2% sodium hypochlorite (NaClO) for 1 min, rinsed with distilled water 3 times, dried on sterile filter paper and placed on 2% potato dextrose agar (PDA) (Crous et al. 2021b). Additionally, ascomata developing on the surface of diseased tissue were gently scraped using a sterile scalpel. Subsequently, a small number of ascospores were transferred and evenly spread on to the surface of a water agar (WA) plate. Hyphal tips originating from leaf tissue fragments and single germinating conidia were transferred on to PDA medium. They were then incubated at 30 °C in darkness (Polizzi et al. 2009). After a period of 7 days, the isolates were transferred on to PDA slants and preserved at 4 °C in the culture collection of Lingnan Normal University. Additionally, they were deposited in the Guangdong Microbial Culture Collection Center (**GDMCC**) in Guangzhou, China. The holotype specimen was preserved in the Herbarium of the Chinese Academy of Forestry (**CAF**) in Beijing, China.

### ﻿Morphological characterisation

Colonies were subcultured on 2% malt extract agar (MEA) and oatmeal agar (OA) at 30 °C for 10 days in the dark (Crous et al. 2009). Colony colour was characterised using Rayner’s Mycological Color Chart (Rayner 1970) and colony diameters were measured after incubation for 10 days at 30 °C in the dark. Morphological characters of ascomata, asci, ascospores, sporodochia, hyphae, conidiomata, conidiophores, conidiogenous cells and conidia were determined in sterile water using an Olympus BX53 compound microscope (Tokyo, Japan), equipped with cellSens Dimension software (version 1.17).

### ﻿DNA extraction, PCR amplification and sequencing

Fungal genomic DNA was extracted from mycelia grown on PDA medium after 10 days using the ENZA Fungal DNA Miniprep Kit (Omega Bio-tek, Doraville, Norcross, GA, U.S.A.), according to the protocol of manufacturer. Four loci, including internal transcribed spacer (ITS) rDNA region, large subunit ribosomal acid (LSU) rDNA region, RNA polymerase II second largest subunit gene (*rpb2*) and part of the beta-tubulin gene (*tub2*), were amplified by the following primer pairs: ITS1 and ITS4 for ITS (White et al. 1990), LR0R and LR5 for LSU (Vilgalys and Hester 1990), RPB150F (Jewell and Hsiang 2013) and fRPB2-7Cr (Liu et al. 1999) and Btub526F and Btub1332R (Jewell and Hsiang 2013). The polymerase chain reaction (PCR) conditions were as follows: 94 °C for 5 min; 94 °C for 30 s, annealing temperature for 45 s and 72 °C for 1 min, 35 cycles; and a final extension step at 72 °C for 10 min. The annealing temperature for ITS and LSU was 54 °C, for *tub2* was 55 °C and for *rpb2* was 57 °C. PCR products were sequenced by Sangon Biotech Co., Ltd. (Shanghai, China). Sequences were edited and assembled using DNAMAN version 5.2.2 and deposited in the NCBI GenBank nucleotide database (Table 1).

**Table 1.** Strains included in the phylogenetic analyses with collection details and GenBank accession numbers.

Species	Voucher	Country	GenBank Accession Number			
			LSU	ITS	*tub2*	*rpb2*
*Microdochiumalbescens*	CBS 290.79	Ivory Coast	KP858950	KP859014	KP859078	KP859123
	CBS 291.79	Ivory Coast	KP858932	KP858996	KP859059	KP859105
	CBS 243.83	Unknown country	KP858930	KP858994	KP859057	KP859103
*M.bolleyi*	CBS 172.63	Germany	MH869857	MH858255	–	–
	CBS 540.92	Syria	KP858946	KP859010	KP859073	KP859119
	Kaz_Mb01	Kazakhstan	–	MW301448	–	–
	Kaz_Mb02	Kazakhstan	–	MW301449	–	–
	CBS 137.64	Netherlands	MH870023	MH858394	–	–
	CPC 25994	Canada	KP858954	KP859018	KP859074	KP859127
	CBS 102891	Germany	MH874405	–	–	–
	CBS 618.72	Germany	MH872294	MH860598	–	–
*M.chrysanthemoides*	CGMCC 3.17929^T^	China	KU746736	KU746690	KU746781	–
	CGMCC 3.17930	China	KU746735	KU746689	KU746782	–
*M.chuxiongense*	YFCC 8794^T^	China	OK586160	OK586161	OK556901	OK584019
*M.citrinidiscum*	CBS 109067^T^	Peru	KP858939	KP859003	KP859066	KP859112
*M.colombiense*	CBS 624.94^T^	Colombia	KP858935	KP858999	KP859062	KP859108
*M.dawsoniorum*	BRIP 65649^T^	Australia	ON394569	MK966337	–	–
	BRIP 67439	Australia	OM333563	MN492650	–	ON624208
*M.fisheri*	CBS 242.90^T^	UK	KP858951	KP859015	KP859079	KP859124
	NFCCI 4083	India	KY777594	KY777595	–	–
	C30 ITI	Sri Lanka	–	MT875317	–	–
*M.graminearum*	CGMCC 3.23525^T^	China	OP104016	OP103966	OP236029	OP236026
	CGMCC 3.23524	China	OP104015	OP103965	OP242835	OP236026
*M.hainanense*	SAUCC210781^T^	China	OM959323	OM956295	OM981146	OM981153
	SAUCC210782	China	OM959324	OM956296	OM981147	OM981154
*M.indocalami*	SAUCC1016^T^	China	MT199878	MT199884	MT435653	MT510550
*M.insulare*	BRIP 75114a	Australia	OQ892168	OQ917075	–	OQ889560
*M.lycopodinum*	CBS 146.68	The Netherlands	KP858929	KP858993	KP859056	KP859102
	CBS 109397	Germany	KP858940	KP859004	KP859067	KP859113
	CBS 109398	Germany	KP858941	KP859005	KP859068	KP859114
	CBS 109399	Germany	KP858942	KP859006	KP859069	KP859115
	CBS 125585^T^	Austria	KP858952	KP859016	KP859080	KP859125
*M.maculosum*	COAD 3358^T^	Brazil	OK966953	OK966954	–	OL310501
*M.majus*	CBS 741.79	Germany	KP858937	KP859001	KP859064	KP859110
	10099	France	–	JX280597	JX280563	JX280560
	10098	France	–	–	JX280564	JX280561
	99027	Canada	–	JX280583	JX280566	–
	200107	Norway	–	KT736191	KT736253	KT736287
*M.miscanthi*	SAUCC211092^T^	China	OM957532	OM956214	OM981141	OM981148
	SAUCC211093	China	OM957533	OM956215	OM981142	OM981149
	SAUCC211094	China	OM957534	OM956216	OM981143	OM981150
*M.musae*	CBS 143500^T^	Malaysia	MH107942	MH107895	MH108041	MH108003
	CBS 143499	Malaysia	MH107941	MH107894	MH108040	–
*M.musae*	CBS 111018	Costa Rica	–	AY293061	–	–
	CPC 11240	Mauritius	MH107944	MH107897	MH108043	–
	CPC 16258	Mexico	MH107945	MH107898	MH108044	–
	CPC 11234	Mauritius	MH107943	MH107896	MH108042	–
	CPC 32681	Malaysia	MH107946	MH107899	–	–
*M.neoqueenslandicum*	CBS445.95	The Netherlands	KP858933	KP858997	KP859060	KP859106
	CBS108926^T^	New Zealand	KP858938	KP859002	KP859065	KP859111
*M.nivale*	CBS 116205^T^	UK	KP858944	KP859008	KP859071	KP859117
	200114	Norway	–	KT736185	–	KT736279
	200119	Norway	–	KT736199	KT736240	KT736263
	200120	Norway	–	KT736210	KT736221	KT736273
	200566	Norway	–	KT736220	KT736224	–
	201050	Norway	–	KT736217	KT736236	KT736257
*M.novae-zelandiae*	CBS 143847	New Zealand	–	LT990655	LT990608	LT990641
	CPC 29693	New Zealand	–	LT990656	LT990609	LT990642
*M.paspali*	CBS 138620^T^	China	–	KJ569509	KJ569514	–
	CBS 138621	China	–	KJ569510	KJ569515	–
	CBS 138622	China	–	KJ569511	KJ569516	–
*M.phragmitis*	CBS 285.71^T^	Poland	KP858949	KP859013	KP859077	KP859122
	CBS 423.78	Germany	KP858948	KP859012	KP859076	KP859121
*M.poae*	CGMCC3.19170^T^	China	–	MH740898	MH740914	MH740906
	LC12115	China	–	MH740901	MH740917	MH740909
	LC12116	China	–	MH740902	MH740918	MH740910
	LC12117	China	–	MH740903	MH740919	MH740911
	LC12118	China	–	MH740897	MH740913	MH740905
	LC12119	China	–	MH740899	MH740915	MH740907
	LC12120	China	–	MH740904	MH740920	MH740912
	LC12121	China	–	MH740900	MH740916	MH740908
*M.ratticaudae*	BRIP 68298^T^	Australia	MW481666	MW481661	–	MW626890
*M.rhopalostylidis*	CBS 145125^T^	New Zealand	MK442532	MK442592	MK442735	MK442667
*M.salmonicolor*	NC14-294	South Korea	MK836108	MK836110	–	–
*M.seminicola*	CBS 122706	Switzerland	KP858943	KP859007	KP859070	KP859116
	CBS 122707	Switzerland	KP858947	KP859011	KP859081	KP859120
	CBS 139951^T^	Switzerland	KP858974	KP859038	KP859101	KP859147
	KAS1516	Canada	KP858961	KP859025	KP859088	KP859134
	KAS3574	Switzerland	KP858973	KP859037	KP859100	KP859146
	KAS3158	Canada	KP858970	KP859034	KP859097	KP859143
	KAS1527	Canada	KP858966	KP859030	KP859093	KP859139
	KAS1473	Canada	KP858955	KP859019	KP859082	KP859128
*M.shilinense*	CGMCC 3.23531^T^	China	OP104022	OP103972	OP242834	–
*M.sinense*	SAUCC211097^T^	China	OM959225	OM956289	OM981144	OM981151
	SAUCC211098	China	OM959226	OM956290	OM981145	OM981152
*M.sorghi*	CBS 691.96	Cuba	KP858936	KP859000	KP859063	KP859109
*Microdochium* sp.	SAUCC1017	China	MT199879	MT199885	MT435654	–
*M.tainanense*	CBS 269.76^T^	Taiwan	KP858945	KP859009	KP859072	KP859118
	CBS 270.76	Taiwan	KP858931	KP858995	KP859058	KP859104
*M.trichocladiopsis*	CBS 623.77^T^	Unknown country	KP858934	KP858998	KP859061	KP859107
*M.triticicola*	RR 241	UK	–	AJ748691	–	–
***M.chrysopogonis***	**GDMCC 3.683**	**China**	**MT988024**	**MT988022**	**MW002441**	**MW002444**
	**LNU-196**	**China**	**MT988023**	**MT988020**	**MW002442**	**MW002445**
	**LNU-197**	**China**	**MT988025**	**MT988021**	**MW002443**	**MW002446**
*M.yunnanense*	SAUCC1018	China	MT199880	MT199886	MT435655	–
	SAUCC1015	China	MT199877	MT199883	MT435652	MT510549
	SAUCC1012	China	MT199876	MT199882	–	MT510548
	SAUCC1011^T^	China	MT199875	MT199881	MT435650	MT510547
*Thamnomycesdendroidea*	CBS 123578	France	KY610467	FN428831	KY624313	KY624232

Note: “T” denotes ex-type strain. Newly-generated sequences are indicated in bold. “-” means no data available in GenBank.

### ﻿Phylogenetic analyses

The sequences of the strains from *C.zizanioides* and those of *Microdochium* species, as well as the outgroup *Idriellalunata* obtained from NCBI GenBank, were aligned with MAFFT version 7 using the default settings. Manual adjustments were made to optimise the alignment in MEGA version 7.0 (Katoh and Standley 2013; Kumar et al. 2016). To elucidate the taxonomic phylogenetic relationships, single and concatenated ITS, LSU, *rpb2* and *tub2* sequence alignments were subjected to analysis by applying Bayesian Inference (BI) using MrBayes version 3.2.5 and Maximum Likelihood (ML) using RAxML on the CIPRES portal (www.phylo.org) (Swofford 2002; Crous et al. 2006; Ronquist et al. 2012). For BI analysis, the best evolutionary model was determined through the utilisation of MrModelTest version 2.2 (Nylander 2004). Subsequently, in MrBayes v. 3.2.5, the Markov Chain Monte Carlo 180 (MCMC) algorithm was used to generate phylogenetic trees. The first 25% of saved trees were discarded as the burn-in phase. Posterior probabilities (PPs) were determined from the remaining trees prior to calculation of the 50% majority rule consensus trees; PP values exceeding 0.90 were considered significant. The ML analyses were performed by using RAxML-HPC BlackBox version 8.2.6, based on 1000 bootstrap replicates. A general time reversible (GTR) model was applied with gamma-distributed rate variation. Bootstrap values (BSs) equal to or higher than 70% were regarded as significant. The phylogenetic tree was viewed in FigTree version 1.4.4 (Rambaut 2018) and edited by Adobe Illustrator CC2018.

### ﻿Pathogenicity test

Three isolates of *M.chrysopogonis* (GDMCC 3.683, LNU-196 and LNU-197) were used to conduct the pathogenicity test. *C.zizanioides* plants were cultivated within a greenhouse, utilising plastic pots containing field-collected soil from the location where the plants had been established. The isolates were cultured on PDA for 2 weeks at 30 °C in the dark to collect conidia.

For the detached leaf assay, 1-cm wide leaves were harvested from 2-month-old plants cultivated in a greenhouse, washed under running tap water, surface disinfected with 70% ethanol for 1 min, rinsed with sterile water for 30 seconds and finally air-dried on sterilised filter paper. The conidial suspension was adjusted to a concentration of 1 × 10^6^ conidia/ml in sterile distilled water. An equivalent volume of sterile distilled water was used as a control. Leaf blades were then wounded with a sterilised pin and each leaf was sprayed with 2 ml of conidial suspension. All inoculated and control leaves were placed in a moist chamber at 25 °C with 100% relative humidity (RH) under cool fluorescent light with a 12-h photoperiod. After seven days, the disease incidence was assessed and calculated as the percentage of leaves with leaf tar spot symptoms. Each treatment consisted of five replicates and the experiment was conducted three times.

For the attached leaf assay, the leaf blades of healthy leaves were also pin-pricked and the conidial suspension was adjusted to a concentration of 2 × 10^6^ conidia/ml in sterile distilled water. An equivalent volume of sterile distilled water was used as a control. In each treatment, five plants were included, with each plant being sprayed with approximately 20 ml of inoculum. All sprayed and control plants were incubated in a plastic container in a greenhouse at 25 ± 2 °C under cool fluorescent light with a 12-h photoperiod. For the first 3 days, the plastic container was covered with transparent polyethylene bags to maintain a high humidity. The disease incidence was assessed 10 days post inoculation and calculated as the percentage of plants displaying tar spot symptoms. Each treatment had three replicates and the pathogenicity test was repeated twice.

To fulfil Koch’s postulates, symptomatic leaf tissues were subjected to surface sterilisation as described above. Subsequently, these tissues were plated on to PDA medium to enable the re-isolation of the fungi. These isolates were identified, based on comparison of the cultures with those of the original strains. Furthermore, the identifications were confirmed by sequencing of the isolates.

### ﻿Effect of temperature on mycelial growth rate

Mycelial growth rates of *M.chrysopogonis* isolates were assessed across various temperatures. Mycelial plugs with a diameter of 5 mm were excised using a sterile hole puncher from the periphery of 10-day-old PDA cultures. Subsequently, they were translocated to the central area of 90 mm PDA Petri dishes. The cultures were subjected to incubation across a temperature range of 5, 10, 15, 20, 25, 30, 35, 40 and 45 °C. Four replicate plates per isolate were prepared for each temperature. The plates were enveloped using Parafilm (Bemis Company, Neenah, WI, U.S.A.) and then positioned within plastic containers prior to their placement in incubators. The colony diameter was measured along two mutually perpendicular axes and the mean of these two measurements was documented as the radial colony diameter. Following a 10-day duration, mycelial growth rates were determined, based on colony diameter and subsequently quantified in millimetres per day. Each treatment was replicated four times.

### ﻿Fungicide sensitivity

To determine possible control measures for this pathogen in the field, six groups including nine fungicides were tested for their ability to inhibit the growth of *M.chrysopogonis* in vitro. Fungicide sensitivity assays were conducted, based on methods developed by Yin et al. (2019). The commercial formulations of fungicides were serially diluted using sterilised distilled water. These diluted solutions were added to autoclaved PDA medium that had been cooled to 55 °C to obtain the desired concentrations in micrograms per millilitre (Table 2). Isolates were cultured on PDA plates at 30 °C for 10 days in darkness to supply inoculum. Mycelial discs (5 mm in diameter) from the periphery of colonies actively growing on PDA were positioned at the centre of the fungicide-amended plates and unamended (control) plates. The plates were then incubated at a temperature of 30 °C in darkness for a duration of 2 weeks. Subsequently, the diameter of each colony was measured along two perpendicular axes and the mean diameter was recalibrated by deducting the diameter of the original plug utilised for inoculation. The effective concentration for 50% mycelial growth inhibition (EC_50_) was estimated by performing a regression analysis of the percentage of mycelial growth inhibition against the log_10_ of fungicide concentrations. Each treatment was replicated four times.

**Table 2.** List of the fungicides used in this study.

Active ingredient	Chemical family	Trade name	FRAC code	Concentration (µg/ml)
Pyrimethanil	anilino-pyrimidines	Syngenta	9	0, 0.01, 0.1, 1, 10, 100
Difenoconazole	triazoles	Syngenta	3	0, 0.01, 0.1, 1, 10, 100
Fludioxonil	phenylpyrroles	Syngenta	12	0, 1, 10, 30, 100, 300
Iprodione	dicarboximides	Syngenta	2	0, 1, 10, 30, 100, 300
Flusilazole	triazoles	Syngenta	3	0, 0.001, 0.01, 0.1, 1, 10
Propiconazole	triazoles	BASF	3	0, 0.0016, 0.008, 0.04, 0.2, 1
Carbendazim	benzimidazoles	Syngenta	1	0, 0.0016, 0.008, 0.04, 0.2, 1
Metalaxyl	acylalanines	BASF	4	0, 10, 30, 100, 300, 1000
Tebuconazole	triazoles	Bayer	3	0, 0.0016, 0.008, 0.04, 0.2, 1

### ﻿Statistical analysis

The dataset was tested for variance homogeneity using the Levene test. If the variances were equal, an analysis of variance (ANOVA) followed by a least significant difference (LSD) test was conducted. In cases where the variances were unequal, the Dunnett T3 test was applied. All statistical analyses were carried out using IBM SPSS version 20.0 (SPSS Inc., Chicago, IL, U.S.A.). The significance threshold for detecting treatment disparities was set at *P* < 0.05.

## ﻿Results

### ﻿Disease symptoms and isolation of the pathogen

**Figure 1.**
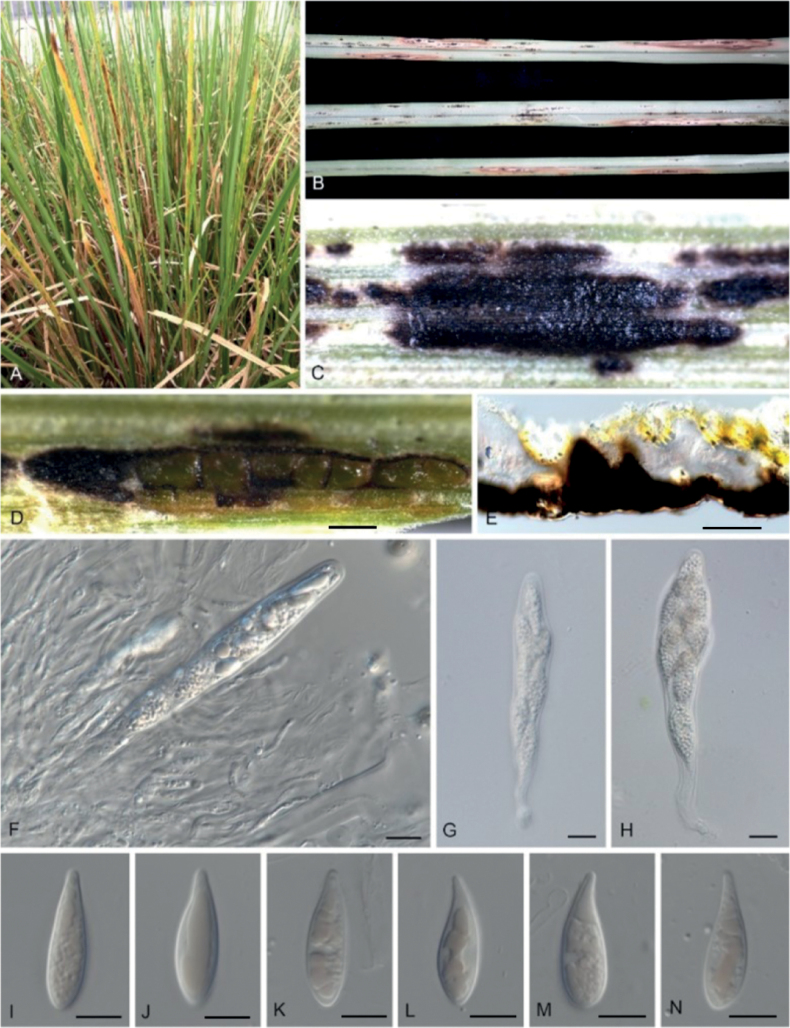
Disease symptoms and morphological characters of *Microdochiumchrysopogonis* on infected leaf tissue (CAF 800053) **A–C** tar spot symptoms of *Chrysopogonzizanioides* from natural infection in the field **D** appearance of immersed ascomata on infected leaves **E** ascomata in longitudinal section **F–H** asci **I–N** ascospores. Scale bars: 100 μm (**D, E**); 10 μm (**F–H**).

From 2019 to 2022, a previously unknown disease of vetiver grass occurred during late spring and early autumn at the Grass Research Station of Lingnan Normal University (LNU) in Guangdong Province, China. Symptoms consistently appeared on 85% of *C.zizanioides* grown under field conditions. The initial symptoms appeared as small and scattered punctate spots (< 1 cm) embedded within the leaf tissue. Gradually, these spots clustered on leaf surfaces. Subsequently, brown, elliptical, fish-eye necrotic haloes emerged, encircling the lesion spots and aligning parallel to the leaf veins (Fig. 1A–C). As these necrotic haloes coalesced, the leaf underwent chlorosis and wilting, eventually leading to blighting of the entire plant. Ascomata were visible on the diseased leaf surfaces.

A total of 67 isolates were obtained on PDA. As the colony morphology of the isolates was consistent, three representative isolates (GDMCC 3.683, LNU-196 and LNU-197), one from each field, were selected for further studies.

### ﻿Phylogeny

Based on a Megablast search on NCBI’s GenBank nucleotide database, the closest hits for the ITS sequence of strain GDMCC 3.683 were *M.dawsoniorum* sequences with 98% identity (538/551, MK966337; 532/543, MN492650) and a *Microdochium* sp. sequence with 97% identity (545/562, FJ536210). The closest hits for LSU sequence of this strain were *M.dawsoniorum* sequences with 99% identity (868/871, OM333563; 864/867, ON394569) and a *M.yunnanense* sequence with 99% identity (875/882, MT199880). The closest hits for its *rpb2* sequence were *M.tainanense* sequences with 85% identity (711/841, KP859118 and KP859104) and a *M.neoqueenslandicum* sequence with 83% identity (698/842, KP859106). The closest hits for *tub2* sequence were a *M.tainanense* sequence with 95% identity (661/697, KP859058), a *M.neoqueenslandicum* sequence with 95% identity (665/703, KP859060) and a *M.colombiense* sequence with 95% identity (658/695, KP859062). Therefore, molecular analyses with all available *Microdochium* species were performed. The alignment of each single locus and concatenated sequence dataset of ITS, LSU, *rpb2* and *tub2* were used to confirm species resolution in *Microdochium*.

**Figure 2.**
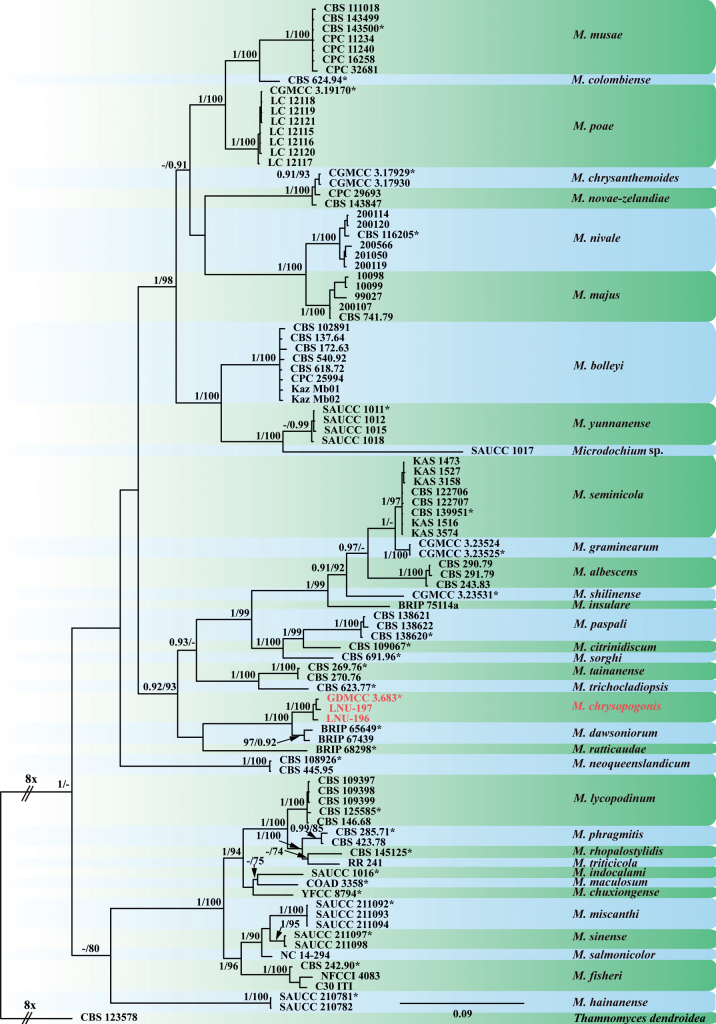
Phylogenetic tree inferred from a Maximum Likelihood analysis, based on a combined alignment of ITS, LSU, *tub2* and *rpb2* sequences from 99 isolates of *Microdochium* sp. Bootstrap support values obtained with ML above 70% and Bayesian (BI) posterior probability values above 0.90 are shown at the nodes (BI/ML). The tree was rooted to *Thamnomycesdendroidea* CBS 123578. Numbers of ex-type strains are emphasised with an asterisk and species are delimited with shaded blocks. Isolates of *M.chrysopogonis* are indicated with lighter text.

There were in total 99 aligned sequences, including the outgroup, *Thamnomycesdendroidea*. A total of 3,033 characters (547 bp from the ITS, 843 bp from LSU, 848 bp from *tub2* and 795 bp from *rpb2*) were included in the phylogenetic analyses. RAxML analysis of the combined dataset yielded a best scoring tree with a final ML optimisation likelihood value of -21, 329.537402 (ln). The matrix had 1,096 distinct alignment patterns with 27.41% undetermined characters or gaps. The tree length was 3.410120. Estimated base frequencies were: A = 0.234382, C = 0.267827, G = 0.258835, T = 0.238956; substitution rates were AC = 1.101009, AG = 4.781387, AT = 1.240884, CG = 0.955029, CT = 6.933148 and GT = 1.000000; gamma distribution shape parameter α = 0.152657. Based on the results of MrModelTest, the SYM + I + gamma for ITS, GTR + I + gamma for LSU and *rpb2* and HKY + I + gamma model for *tub2* were selected as the best fit models for Bayesian analyses. A total of 47,402 trees were generated by BI, amongst which 11,851 trees were discarded as the burn-in phase and the remaining 35,551 trees were used to calculate the posterior probabilities (PPs). The BI consensus tree confirmed the tree topology obtained with ML. The well-supported clade (1/100) formed by the three strains from *C.zizanioides* clustered with high support (1/100) with *M.dawsoniorum* (0.92/97), which was sister to one single-strain clade representing *M.ratticaude*. This clade clustered with high support (0.92/93) with the clade formed by *M.albescens*, *M.seminicola*, *M.graminearum*, *M.shilinense*, *M.insulare*, *M.paspali*, *M.citrinidiscum*, *M.sorghi*, *M.tainanense* and *M.trichocladiopsis* strains. The *M.neoqueenslandicum* clade (1/100) was basal to this clade (Fig. 2). Single gene-based phylogenies are presented in the Suppl. material 1. Nonetheless, these individual gene trees did not yield a conclusive taxonomic classification for the new species, in contrast to the comprehensive resolution achieved through the concatenated sequence analysis.

## ﻿Taxonomy

Based on multilocus phylogenetic analyses, the three strains isolated from *C.zizanioides* represent a previously unknown species within the genus *Microdochium* that is closely related to *M.dawsoniorum* and *M.ratticaudae*. Morphological data placed the new species in the genus *Microdochium*. This species is characterised below.

### ﻿ Microdochium chrysopogonis

Taxon classificationFungiAmphisphaerialesAmphisphaeriaceae

W. Zhang & X. Lu sp. nov.

80780F57-2487-5F75-8EFC-DD131E495D5B

845624

Figs 1 , 3

#### Etymology.

Name refers to *Chrysopogon*, the host genus from which this fungus was collected.

#### Description.

***Sexual morph*** on infected leaf tissue of the host plant (CAF 800054). ***Ascomata*** perithecial, 300–350 μm diam., solitary or in groups, immersed, pale brown to black, subglobose to oval, uniloculate, non-ostiolate. ***Paraphyses*** filiform, hyaline, straight or curved, apically free. ***Asci*** 50–60 × 10–18, *x*¯ = 55 × 13 μm (n = 50), hyaline, fasciculate, unitunicate, oblong to narrowly clavate, fusiform, 8 biseriate spores with a short stipe. ***Ascospores*** clavate, hyaline, guttulate, 20–22 × 8–11.5, *x*¯ = 21 × 9 μm (n = 50), aseptate, smooth. ***Sporodochia*** salmon-pink, slimy. ***Conidiophores*** reduced to conidiogenous cells. ***Conidiogenous cells*** with percurrent proliferation, hyaline, smooth, aseptate, ampulliform or obpyriform, 10–23 × 8–11.5, *x*¯ = 17 × 9.5 μm (n = 50). ***Conidia*** fusiform, lunate, curved, solitary, guttulate, variable in length, 0–1-septate, 18–72 × 2–3.5, *x*¯ = 38.5 × 3 μm (n = 50), apex rounded, base usually flattened. ***Chlamydospores*** not observed. Vegetative hyphae on PDA (GDMCC 3.683) superficial and immersed, septate, branched, hyaline, smooth, 1–5.5 μm wide.

#### Culture characteristics.

Colonies on PDA reaching 4.0–4.5 cm within seven days in the dark at 30 °C, flat, white cottony aerial mycelium, dense, saffron rounded sporodochia produced after 3 weeks; reverse saffron. On MEA, sparse white cottony aerial mycelium, orange rounded sporodochia produced; reverse salmon-pink. On OA, periphery with white scarce cottony aerial mycelium, concentric rings of orange rounded sporodochia produced; reverse orange.

#### Type.

China, Guangdong Province, Zhanjiang City, field of the Grass Research Station of Lingnan Normal University (LNU), from a leaf of vetiver grass (*Chrysopogonzizanioides*) with leaf tar spot disease, September 2019, W. Zhang & X. Lu, holotype CAF 800054, ex-type living strain GDMCC 3.683.

#### Additional materials examined.

China, Guangdong Province, Zhanjiang City, field of the Grass Research Station of Lingnan Normal University (LNU), from a leaf of vetiver grass (*C.zizanioides*) with leaf tar spot disease, September 2019, W. Zhang & X. Lu, strain LNU-196; China, Guangdong Province, Zhanjiang City, field of the Grass Research Station of Lingnan Normal University (LNU), from a leaf of vetiver grass (*C.zizanioides*) with leaf tar spot disease, September 2019, W. Zhang & X. Lu, strain LNU-197.

#### Notes.

A multilocus phylogenetic analysis of the ITS, LSU, *tub2* and *rpb2* loci placed three strains of *M.chrysopogonis* in a distinct and monophyletic clade (1/100) sister to *M.dawsoniorum* and *M.ratticaudae*. Notably, *M.chrysopogonis* has longer conidia (18–72 × 2–3.5 μm) than *M.ratticaudae* (7–11 × 1.5–2.5 μm) and wider conidia than *M.dawsoniorum* (25–75 × 1–2 μm). Furthermore, the conidia of *M.chrysopogonis* are guttulate and 0–1-septate, while those of *M.dawsoniorum* are 0–3-septate and those of *M.ratticaudae* are aseptate. The conidiogenous cells of *M.chrysopogonis* appear as percurrent, ampulliform or obpyriform, whereas those of *M.ratticaudae* are indistinct from the hyphae and those of *M.dawsoniorum* are cylindrical to irregular and flexuous. Additionally, the conidiogenous cells of *M.chrysopogonis* (10–23 × 8–11.5 μm) are wider than those of *M.ratticaudae* (20–30 × 1–2 μm) (Table 3). Differences are also evident in the sexual morph of these three species. In particular, the sexual morph is not observed in *M.dawsoniorum*. Ascomata size varies, with that of *M.ratticaudae* (100–160 μm) being smaller than that of *M.chrysopogonis* (300–350 μm). Ascospores of *M.ratticaudae* (14–24 × 4–7 μm) are fusoid to navicular, while those of *M.chrysopogonis* are clavate, guttulate and wider (20–22 × 8–11.5 μm). In addition, *M.ratticaudae* features abundant, pale to olivaceous brown, subglobose or cylindrical chlamydospores, while these are not observed in *M.chrysopogonis* (Crous et al. 2020, 2021a; Table 3). Consequently, based on both morphological characteristics and phylogenetic analyses, all three isolates of *M.chrysopogonis* were proposed as a new species.

**Figure 3.**
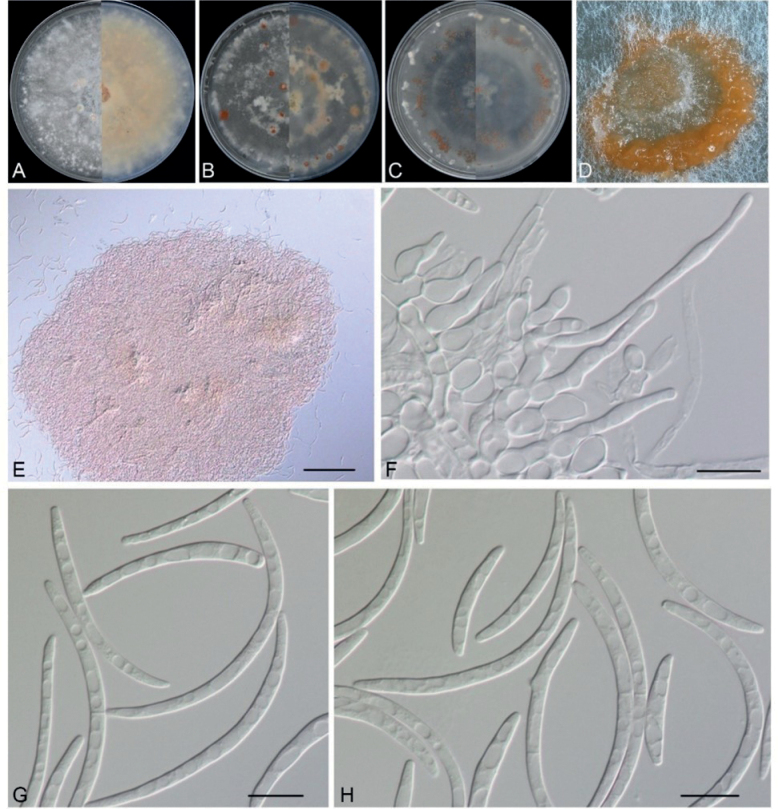
*Microdochiumchrysopogonis* (from ex-type: GDMCC 3.683) **A** colonies after 7 days on PDA**B** colonies after 7 days on MEA**C** colonies after 7 days on OA**D** colony overview of the sporodochia on PDA in culture after incubation for three weeks **E** aggregated conidiophores **F** conidiophores with conidiogenous cells **G, H** conidia. Scale bars: 20 μm (**B**).

**Table 3.** Morphological characters of *Microdochiumchrysopogonis* and its related species.

Taxa			*M.albescens*	*M.citrinidiscum*	*M.neoqueenslandicum*	*M.paspali*	*M.seminicola*	*M.trichocladiopsis*	*M.tainanense*	*M.sorghi*	*M.dawsoniorum*	*M.ratticaudae*	*M.graminearum*	*M.shilinense*	*M.chrysopogonis*
Asexual morph	Conidia	Shape	falcate, slightly to strongly curved, apex pointed	cylindrical, clavate, obovoid	lunate, allantoid, curved	falcate, apex pointed	cylindrical to fusiform, straight or curved	oblong, fusiform to obovoid, straight or curved	lunate	filiform, narrowly acicular fusiform, obclavate	flexuous to falcate, sometimes with a geniculation, acute at the tip, narrow at the base	fusoid, falcate, acute at the apex and narrowed at the base	n/a	n/a	fusiform, lunate, curved, guttulate
		Size (μm)	11–16 × 3.5–4.5	7–31 × 2–3	4–9 × 1.5–3	7–20.5 × 2.5–4.5	19–54 × 3–4.5	6–18 × 2–3.5	10–15 × 2–3	20–90 × 1.5–4.5	25–75 × 1–2	7–11 × 1.5–2.5	n/a	n/a	18–72 × 2–3.5
		Septa	0–1(–3)	0–3	0(–1)	0–3	(0–)3(–5)	0(–1)	0–1	1–7(–10)	0–3	aseptate	n/a	n/a	0–1
	Conidiogenous cells	Shape	subcylindrical, doliiform to obpyriform	denticulate, cylindrical	ampulliform, lageniform to subcylindrical	ampulliform, lageniform to cylindrical	ampulliform to lageniform	cylindrical to clavate, straight but often curved at the tip	sympodial, apical, cylindrical or ampulliform with conspicuous rhachides	sympodial, ovoid, ampulliform to obclavate	cylindrical to irregular, flexuous, narrowed towards the tip	indistinct from hyphae, terminal, solitary.	n/a	n/a	ampulliform or obpyriform
		Size (μm)	6–15 × 1.5–4	11–29 × 1.5–2	4.5–10 × 2–3.5	6.5–15.5 × 2.5–4	7–9.5 × 3–4	4–37 × 2–3	3–10 × 1–3	5–13 × 3–4	20–30 × 1–2	n/a	n/a	16.3–22.4 × 4.1–5.7	10–23 × 8–11.5
Sexual morph	Chlamydospores	Shape	n/a	n/a	n/a	n/a	n/a	present	n/a	n/a	n/a	subglobose or cylindrical	n/a	n/a	n/a
	Perithecia	Size (μm)	150–180 × 90–120	n/a	n/a	n/a	110–149	n/a	n/a	n/a	n/a	100–160	n/a	n/a	300–350
	Asci	Size (μm)	40–85 × 8–12	n/a	n/a	n/a	41–66 × 7.6–11	n/a	n/a	n/a	n/a	50–75 × 10–14	55–77.5 × 9.5–15.	50–76 × 7–10	50–60 × 10–18
	Ascospores	Size (μm)	14–23 × 3.5–4.5	n/a	n/a	n/a	12–22 × 3–4.5	n/a	n/a	n/a	n/a	14–24 × 4–7	16.5–24 × 4–5.5	14–18 × 3–5.5	20–22 × 8–11.5
		Septa	1–3(–5)	n/a	n/a	n/a	0–3	n/a	n/a	n/a	n/a	aseptate	0–3	0–3	aseptate
References			Hernández-Restrepo et al. (2016)	Hernández-Restrepo et al. (2016)	Hernández-Restrepo et al. (2016)	Zhang et al. (2015) (Continued on next page)	Hernández-Restrepo et al. (2016)	Hernández-Restrepo et al. (2016)	De Hoog & Hermanides-Nijhof (1977)	Braun (1995)	Crous et al. (2020) (Continued on next page)	Crous et al. (2021a)	Gao et al. (2022)	Gao et al. (2022)	This study

Note: “n/a” means not provided in the literature.

##### ﻿Pathogenicity test

**Figure 4.**
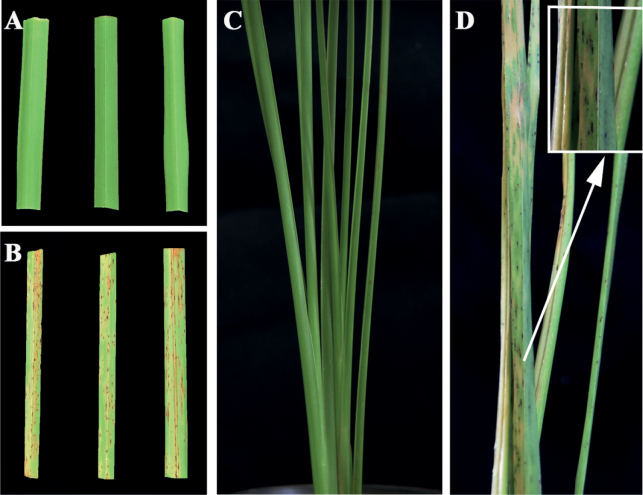
Tar spot symptoms of *Chrysopogonzizanioides* 7 days after spraying on detached leaves (**A, B**) and 10 days after spraying on leaves attached to whole plants (**C, D**) with *Microdochiumchrysopogonis* isolate GDMCC 3.683 (**B, D**) and sterilised water (**A, C**).

**Figure 5.**
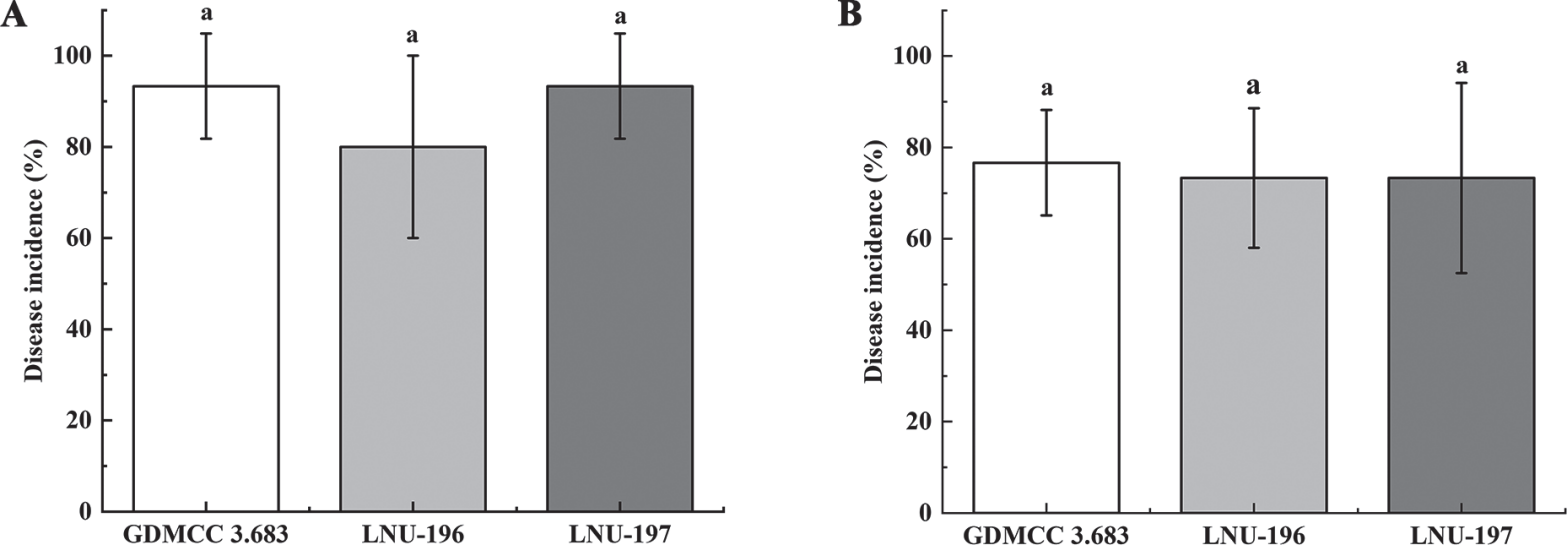
Disease incidence of tar spot symptoms on *Chrysopogonzizanioides* for leaves 7 days after spraying detached leaves (**A**) and for whole plants 10 days after spraying leaves attached to potted plants (**B**), respectively, with *Microdochiumchrysopogonis* isolates GDMCC 3.683, LNU-196 and LNU-197. Values are shown as the means, with the error bars representing the standard error. For each pathogen, columns with the same letter indicate means that are not significantly different according to a least significant difference (LSD) test (*P* < 0.05).

The symptoms observed on leaves of *C.zizanioides* after inoculation with the representative isolate GDMCC 3.683 were similar to those observed in the field. No symptoms were observed on the leaves of the negative controls (Fig. 4). The average disease incidence of detached leaves that were wounded and sprayed with the isolates GDMCC 3.683, LNU-196 and LNU-197 was 93.3%, 80.0% and 93.3%, respectively. The average disease incidence of whole plants after spraying with the same isolates was 76.7%, 73.3% and 73.3%, respectively (Fig. 5). Koch’s postulates were fulfilled by successful re-isolation of the fungal strains from all leaf spot tissues inoculated with the three isolates. The morphology and DNA sequences of the isolates re-isolated from the inoculated tissues were consistent with those of the strains used for inoculations.

##### ﻿Effect of temperature on mycelial growth

**Figure 6.**
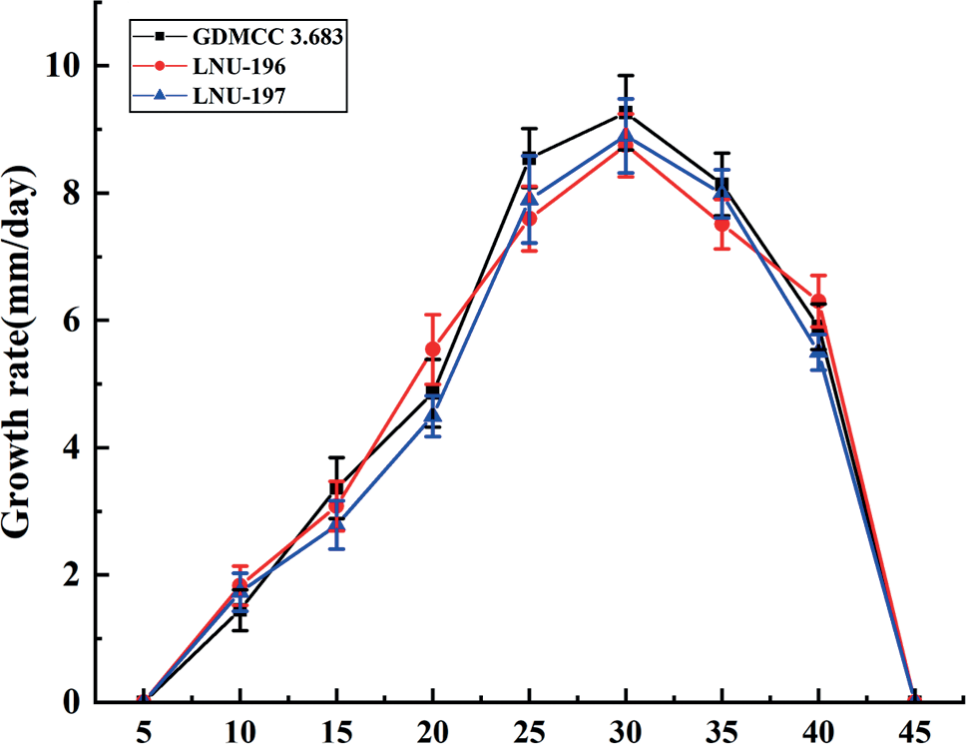
Colony growth rate of three isolates, GDMCC 3.683, LNU-196 and LNU-197, of *Microdochiumchrysopogonis* from *Chrysopogonzizanioides* under different temperatures. Error bars represent the standard error.

The mycelial growth of *M.chrysopogonis* was significantly affected by temperature (*P* < 0.01). All three isolates of *M.chrysopogonis* grew between 10 and 40 °C, with maximum growth observed at 30 °C (Fig. 6). No isolates grew at 5 or 45 °C after 3 days. The highest average mycelial growth rate was observed at 30 °C (26.5 ± 2.0 mm/day), followed by 25 °C (20.1 ± 4.7 mm/day).

##### ﻿Fungicide sensitivity

The EC_50_ values of various fungicides were analysed for their effectiveness against *M.chrysopogonis* isolates. A total of 17 isolates of *M.chrysopogonis* were collected from diseased leaves spanning the period from 2019 to 2022.

**Figure 7.**
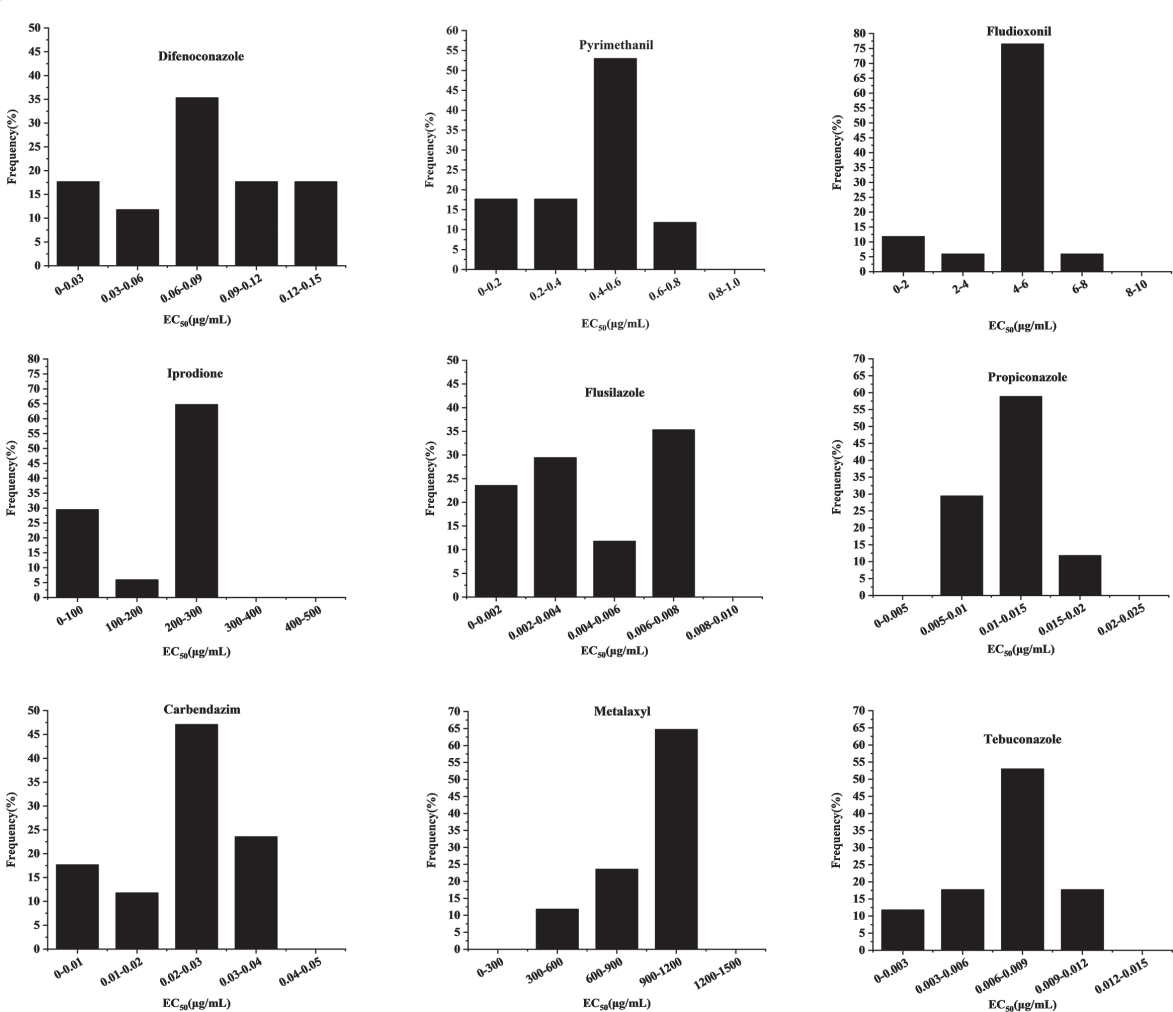
Frequency distribution of the 50% effective concentration (EC_50_) values of six groups including nine fungicides for *Microdochiumchrysopogonis* isolates, based on mycelial growth from 2019 to 2022.

The frequency distribution showed that difenoconazole, fludioxonil, flusilazole, carbendazim and iprodione exhibited distributions resembling normal curves, while pyrimethanil, propiconazole, metalaxyl and tebuconazole displayed unimodal curves (Fig. 7). EC_50_ values for the inhibition of 17 *M.chrysopogonis* isolates, based on mycelial radial growth, varied across fungicide treatments (*P* < 0.05) (Table 4). Amongst the tested fungicides, flusilazole had the lowest EC_50_ values, with a notably concentrated response range of 0.001 to 0.007 µg/ml and an average of 0.004 µg/ml. Tebuconazole closely followed with a slightly narrower range, exhibiting values ranging from 0.002 to 0.009 µg/ml and an average of 0.007 µg/ml. Furthermore, there was no significant difference between flusilazole and tebuconazole. Propiconazole displayed EC_50_ values spanning from 0.006 to 0.016 µg/ml, with an average of 0.011 µg/ml, while those of carbendazim ranged from 0.008 to 0.031 µg/ml, with an average of 0.024 µg/ml. In contrast, those of difenoconazole exhibited a broader range, varying from 0.013 to 0.127 µg/ml, with a mean value of 0.077 µg/ml, while those of pyrimethanil ranged from 0.054 to 0.605 µg/ml, with an average of 0.411 µg/ml. Those of iprodione, on the other hand, spanned from 15.018 to 260.335 µg/ml, with an average of 193.031 µg/ml. Metalaxyl exhibited the highest EC_50_ value, displaying the widest range amongst all fungicides, extending from 302.785 to 1056.896 µg/ml with an average of 892.677 µg/ml. Overall, these findings indicate varying degrees of sensitivity to different fungicides amongst *M.chrysopogonis* isolates. These variations in sensitivity could be essential considerations for designing effective fungicide application strategies against vetiver leaf tar spot disease.

**Table 4.** In vitro sensitivity ranges and mean 50% effective concentration (EC_50_) values for the inhibition of *Microdochiumchrysopogonis*.

Fungicide	EC_50_ (μg/ml)		
	Lowest	Highest	Mean ± SE
Difenoconazole	0.013	0.127	0.077 ± 0.039e
Pyrimethanil	0.054	0.605	0.411 ± 0.180d
Fludioxonil	0.014	6.128	4.525 ± 1.626c
Iprodione	15.018	260.335	193.031 ± 99.462b
Flusilazole	0.001	0.007	0.004 ± 0.003h
Propiconazole	0.006	0.016	0.011 ± 0.003g
Carbendazim	0.008	0.031	0.024 ± 0.009f
Metalaxyl	302.785	1056.896	892.677 ± 236.145a
Tebuconazole	0.002	0.009	0.007 ± 0.002h

Note: The letters indicate the comparison amongst the different fungicide treatments. Means followed by the same letter do not differ according to a post hoc Dunnett T3 test (*p* < 0.05).

**Figure 8.**
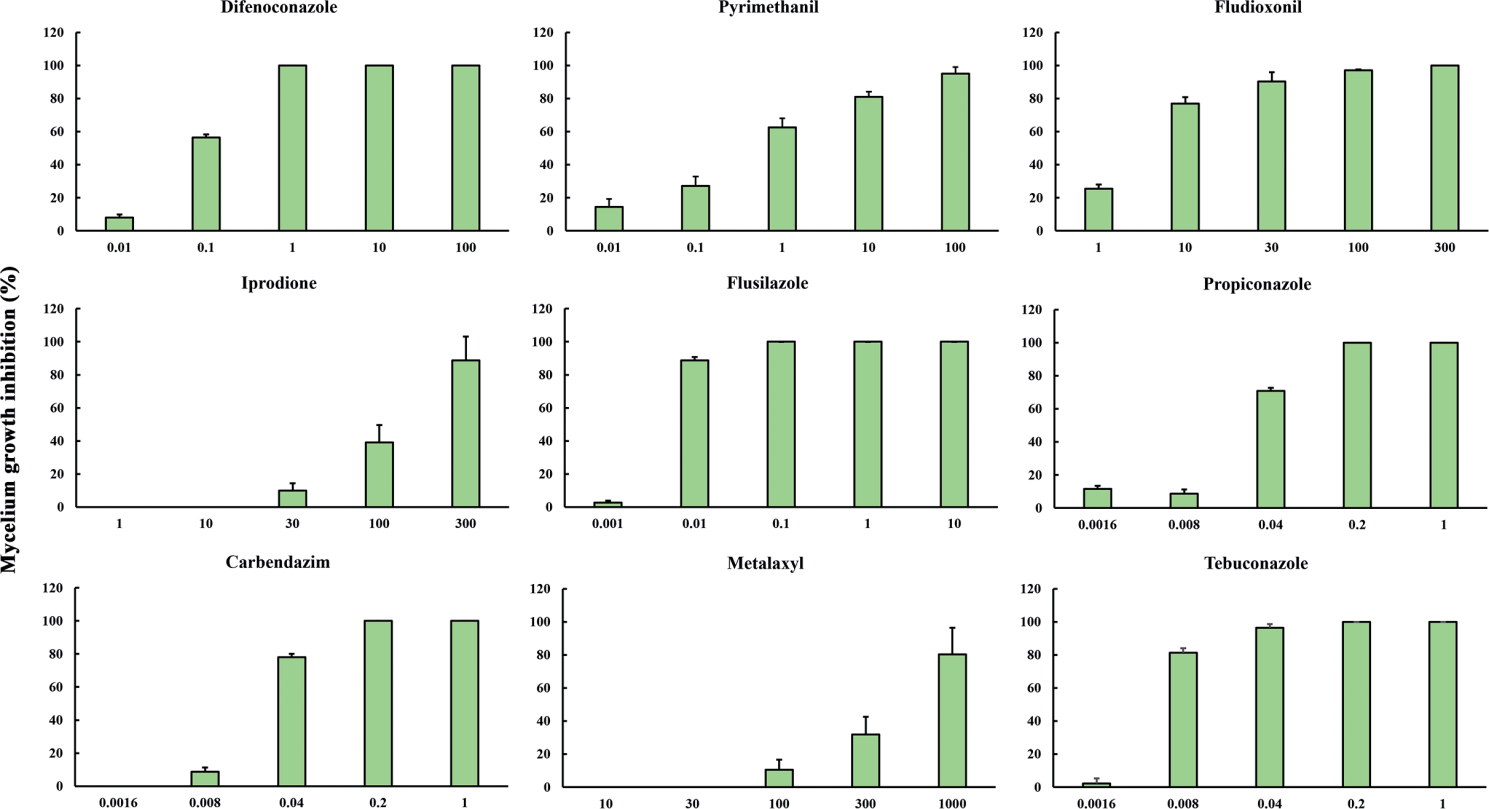
Effect of fungicides on the mycelial growth of *Microdochiumchrysopogonis*. Values are shown as the means, with the error bars representing the standard error.

The inhibition of mycelial growth revealed that all nine fungicides exhibited a reduction in fungal growth in vitro when compared to plates without amendments. The effectiveness of these fungicides in diminishing the mycelial growth of the isolates was contingent upon both the specific chemical compound and its concentration. Four DMI fungicides, namely, difenoconazole, propiconazole, flusilazole and tebuconazole and one MBC fungicide, carbendazim, displayed strong activity against *M.chrysopogonis* growth at concentrations below 1 µg/ml, specifically at concentrations of 1, 0.2, 0.1, 0.2 and 0.2 µg/ml, respectively (Fig. 8). However, *M.chrysopogonis* showed a tendency to exhibit better growth in the presence of pyrimethanil, fludioxonil, iprodione and metalaxyl, with mycelial growth being completely inhibited at concentrations exceeding 100 µg/ml (Fig. 8).

## ﻿Discussion

In a survey of disease on *C.zizanioides* in Guangdong Province, China, from 2019 to 2022, tar spot was the predominant leaf spot disease. Isolation, morphological features, multilocus phylogenetic analysis and pathogenicity tests confirmed that a new *Microdochium* species, *M.chrysopogonis* was the causal agent. To effectively control the disease, the sensitivity of *M.chrysopogonis* to six groups of fungicides, including nine fungicides was determined. Results indicated that four DMI fungicides, namely difenoconazole, propiconazole, flusilazole and tebuconazole and one MBC fungicide, carbendazim, were highly effective against the new species.

The morphology of the new species is introduced along with its sexual and asexual morphological features, which are consistent with the following of *Microdochium*: pale brown to black, subglobose to oval, uniloculate, perithecial ascomata; hyaline, fasciculate, unitunicate, oblong to narrowly clavate, eight biseriate spores with short stipe asci, from which hyaline, clavate, smooth ascospores arise. Conidiophores reduced to hyaline, smooth, aseptate, percurrent, ampulliform or obpyriform, conidiogenous cells, from which hyaline, 0–1-septate, fusiform, lunate conidia with the apex rounded and base flattened usually arise (Figs 1, 3) (Hernández-Restrepo et al. 2016). The concatenated ITS, LSU, *tub2* and *rpb2* sequences were able to identify species in *Microdochium* and proved to be suitable barcoding markers in the process of species resolution (Hernández-Restrepo et al. 2016). Phylogenetic analysis indicated that *M.chrysopogonis* formed a distinct well-supported clade (1/100) and was closely related to *M.dawsoniorum* and *M.ratticaudae* (Fig. 2). Nevertheless, the classification of the new species in the genus *Microdochium* is well supported by morphology, based on sexual and asexual morphs, which are different from those of *M.dawsoniorum* and *M.ratticaudae*.

Temperature is a major factor affecting plant disease epidemics. In recent years, tar spot disease of *C.zizanioides* has become increasingly prevalent in Guangdong Province, China, especially in hot and rainy summers. Thus, the effect of temperature on the growth rate of *M.chrysopogonis* in vitro was evaluated in this study. There were no significant differences in the minimum and optimum growth temperatures amongst the three isolates and the optimum growth temperature was 30 °C (Fig. 6). Research revealed that the highest growth rate of *M.paspali* occurred at 25–28 °C and *M.majus*, *M.seminicola* and *M.nivale* strains in Russia and Europe grew optimally at 20–25 °C (Doohan et al. 2003; Gagkaeva et al. 2020), while *M.nivale* from Slovakia grew better at temperatures below 20 °C (Hudec and Muchová 2010). Thus, the optimum growth temperature varies amongst *Microdochium* species.

A previous study showed that *P.herbarum* could initially induce leaf spots and blight on vetiver grass, causing round or irregular dark brown spots, which are similar to the symptoms on *M.chrysopogonis* (Zhang et al. 2017). However, the symptoms on *M.chrysopogonis* were different from those on *P.herbarum* in the later period. Specifically, *P.herbarum* caused fusiform or irregular with reddish-brown margins on the host plant, whereas *M.chrysopogonis* caused fish-eye necrotic haloes surrounding the spot lesions on leaves. Additionally, the disease incidences were different. *P.herbarum* affected 26% to 42% of plants, while *M.chrysopogonis* showed a 100% disease incidence. Given the high disease incidence associated with *M.chrysopogonis* and its induction of leaf spots on vetiver grass, as well as the identification of this new species, it is imperative to conduct future studies addressing the host spectrum, epidemic conditions, biological characteristics and distribution patterns of *M.chrysopogonis*.

The effectiveness of biofungicides, such as bacterial seed treatments using *Pseudomonas* and *Pantoea* in controlling diseases caused by *Microdochium*, has been established (Johansson et al. 2003). However, there remains substantial reliance on registered chemical fungicides. Currently, research on fungicide sensitivity within *Microdochium* mainly focuses on three species: *M.panattonianum*, *M.majus* and *M.nivale*. Six groups of fungicides, namely, MBCs, DMIs, QoIs, SDHIs, PPs, and dicarboximides, have been shown to have significant inhibitory activity (Kaneko and Ishii 2009; Aamlid et al. 2017, 2018; Matušinsky et al. 2017; Gagkaeva et al. 2022). In this study, consistent with previous findings, four DMI fungicides (difenoconazole, propiconazole, flusilazole and tebuconazole) and one MBC fungicide (carbendazim) exhibited significant inhibitory effects on the growth of *M.chrysopogonis*, with mean EC_50_ values of 0.077, 0.011, 0.004, 0.024 and 0.007 μg/ml, respectively (Table 4). However, dicarboximides (iprodione), which were effective against snow mould and Microdochium patch caused by *M.nivale* on turf-grass in previous studies (Gourlie and Hsiang 2017), showed ineffectiveness in this study, with a mean EC_50_ value of 193.031 μg/ml. Additionally, while the PP fungicide fludioxonil demonstrated good antifungal activity against *M.majus* in other research (Mao et al. 2023), the isolates in this study displayed only moderate sensitivity to fludioxonil, with an EC_50_ value of 4.525 μg/ml and complete inhibition of mycelial growth required concentrations exceeding 100 µg/ml (Table 4, Fig. 8). These variations in fungicide sensitivity could be attributed to genetic structural changes, introducing bias in chemical control efficacy (Matušinsky et al. 2019). Furthermore, the response of the same pathogen to fungicides can vary amongst regions. For example, the DMI fungicides, tebuconazole and metconazole, were reported to be ineffective against *M.nivale* in the Czech Republic and France (Ioos et al. 2005; Matušinsky et al. 2019). Similarly, *M.nivale* exhibited sensitivity to SDHI fungicides, including pydiflumetofen, fluxapyroxad and penthiopyrad, in vitro, but these fungicides were ineffective in providing acceptable control under field conditions in the USA (Hockemeyer and Koch 2022). These differences may be attributed to variations in environmental factors, such as temperature and humidity, as well as diverse biological characteristics, including epidemiology, fungicide sensitivity and aggressive nature of the pathogen (Abdelhalim et al. 2020). Overall, this study offers valuable insights into fungicide application strategies for effectively managing the disease. Further research is needed to analyse the influences of environmental variables and conduct field trials to validate the effects of DMI fungicides, ultimately enhancing the ability to successfully manage vetiver leaf tar spot disease.

## Supplementary Material

XML Treatment for Microdochium chrysopogonis

## ﻿Additional information

### Author ORCIDs

Xiang Lu https://orcid.org/0000-0001-9582-1319

Mengxian Mai https://orcid.org/0009-0001-3824-2895

Wenhui Tan https://orcid.org/0009-0008-6054-2174

Muyan Zhang https://orcid.org/0009-0005-8880-9780

Jie Xie https://orcid.org/0009-0002-4200-0140

Yi Lu https://orcid.org/0009-0003-0848-2390

### Data availability

All of the data that support the findings of this study are available in the main text or Supplementary Information.

## Supplementary materials

Supplementary material 1 Phylogenetic tree inferred from a maximum likelihood analysis based on a combined alignment of ITS sequences of 97 isolates of the *Microdochium* sp. Data type tif Explanation note Bootstrap support values obtained with ML above 70% and Bayesian (BI) posterior probability values above 0.90 are shown at the nodes (BI/ML). The designated outgroup taxa are *Thamnomycesdendroidea* CBS 123578. Numbers of ex-type strains are emphasized with an asterisk and species are delimited with shaded blocks. Isolates of *M.chrysopogonis* are indicated with lighter text. https://binary.pensoft.net/file/948395This dataset is made available under the Open Database License (http://opendatacommons.org/licenses/odbl/1.0/). The Open Database License (ODbL) is a license agreement intended to allow users to freely share, modify, and use this Dataset while maintaining this same freedom for others, provided that the original source and author(s) are credited.Xiang Lu, Mengxian Mai, Wenhui Tan, Muyan Zhang, Jie Xie, Yi Lu, Xue Li Niu, Wu Zhang

Supplementary material 2 Phylogenetic tree inferred from a maximum likelihood analysis based on a combined alignment of LSU sequences of 72 isolates of the *Microdochium* sp. Data type tif Explanation note Bootstrap support values obtained with ML above 70% and Bayesian (BI) posterior probability values above 0.90 are shown at the nodes (BI/ML). The designated outgroup taxa are *Thamnomycesdendroidea* CBS 123578. Numbers of ex-type strains are emphasized with an asterisk and species are delimited with shaded blocks. Isolates of *M.chrysopogonis* are indicated with lighter text. https://binary.pensoft.net/file/948396This dataset is made available under the Open Database License (http://opendatacommons.org/licenses/odbl/1.0/). The Open Database License (ODbL) is a license agreement intended to allow users to freely share, modify, and use this Dataset while maintaining this same freedom for others, provided that the original source and author(s) are credited.Xiang Lu, Mengxian Mai, Wenhui Tan, Muyan Zhang, Jie Xie, Yi Lu, Xue Li Niu, Wu Zhang

Supplementary material 3 Phylogenetic tree inferred from a maximum likelihood analysis based on a combined alignment of *rpb2* sequences of 71 isolates of the *Microdochium* sp. Data type tif Explanation note Bootstrap support values obtained with ML above 70% and Bayesian (BI) posterior probability values above 0.90 are shown at the nodes (BI/ML). The designated outgroup taxa are *Thamnomycesdendroidea* CBS 123578. Numbers of ex-type strains are emphasized with an asterisk and species are delimited with shaded blocks. Isolates of *M.chrysopogonis* are indicated with lighter text. https://binary.pensoft.net/file/948397This dataset is made available under the Open Database License (http://opendatacommons.org/licenses/odbl/1.0/). The Open Database License (ODbL) is a license agreement intended to allow users to freely share, modify, and use this Dataset while maintaining this same freedom for others, provided that the original source and author(s) are credited.Xiang Lu, Mengxian Mai, Wenhui Tan, Muyan Zhang, Jie Xie, Yi Lu, Xue Li Niu, Wu Zhang

Supplementary material 4 Phylogenetic tree inferred from a maximum likelihood analysis based on a combined alignment of *tub2* sequences of 80 isolates of the *Microdochium* sp. Data type tif Explanation note Bootstrap support values obtained with ML above 70% and Bayesian (BI) posterior probability values above 0.90 are shown at the nodes (BI/ML). The designated outgroup taxa are *Thamnomycesdendroidea* CBS 123578. Numbers of ex-type strains are emphasized with an asterisk and species are delimited with shaded blocks. Isolates of *M.chrysopogonis* are indicated with lighter text. https://binary.pensoft.net/file/948398This dataset is made available under the Open Database License (http://opendatacommons.org/licenses/odbl/1.0/). The Open Database License (ODbL) is a license agreement intended to allow users to freely share, modify, and use this Dataset while maintaining this same freedom for others, provided that the original source and author(s) are credited.Xiang Lu, Mengxian Mai, Wenhui Tan, Muyan Zhang, Jie Xie, Yi Lu, Xue Li Niu, Wu Zhang
