# Supplementary figures and images for: ﻿Identification and fungicide sensitivity of Microdochiumchrysopogonis (Ascomycota, Amphisphaeriaceae), a new species causing tar spot of Chrysopogonzizanioides in southern China
Source: MycoKeys. 2023 Dec 6;100:205–32. doi: 10.3897/mycokeys.100.112128 (PMC10719939; doi:10.3897/mycokeys.100.112128)

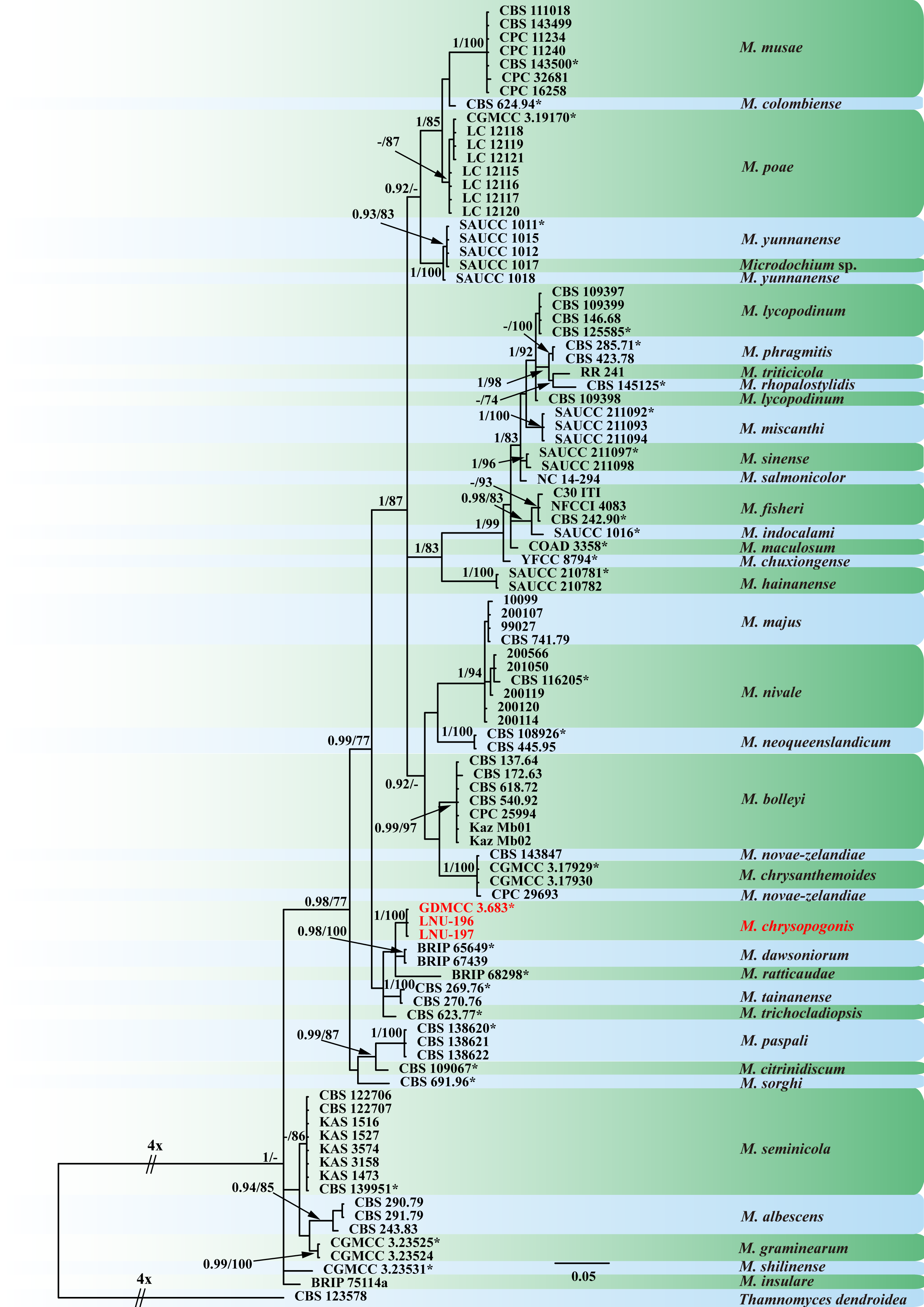

Supplement: Supplementary material 1 — Phylogenetic tree inferred from a maximum likelihood analysis based on a combined alignment of ITS sequences of 97 isolates of the Microdochium sp. [file mycokeys-100-205-s001.tif]

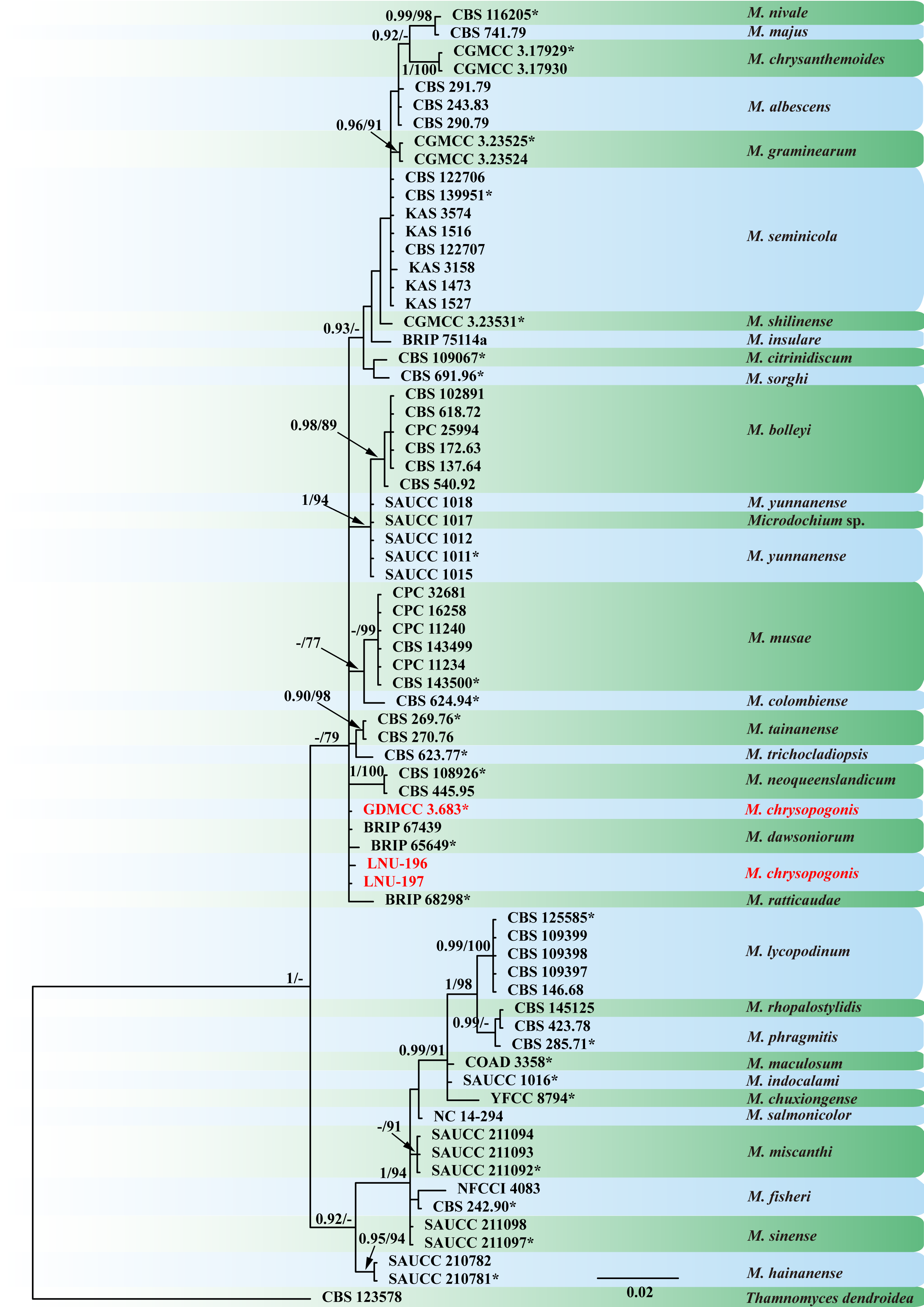

Supplement: Supplementary material 2 — Phylogenetic tree inferred from a maximum likelihood analysis based on a combined alignment of LSU sequences of 72 isolates of the Microdochium sp. [file mycokeys-100-205-s002.tif]

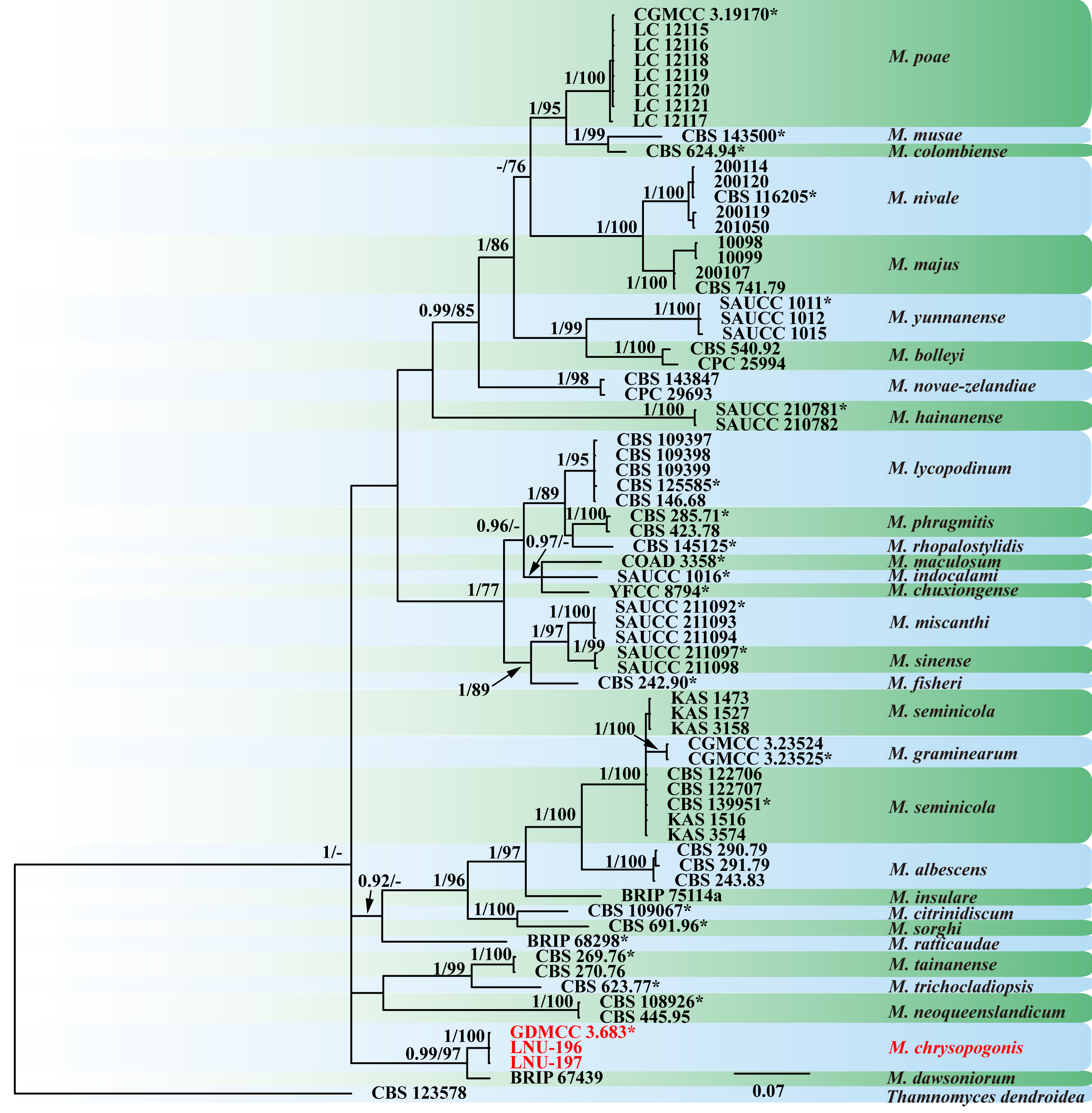

Supplement: Supplementary material 3 — Phylogenetic tree inferred from a maximum likelihood analysis based on a combined alignment of rpb2 sequences of 71 isolates of the Microdochium sp. [file mycokeys-100-205-s003.tif]

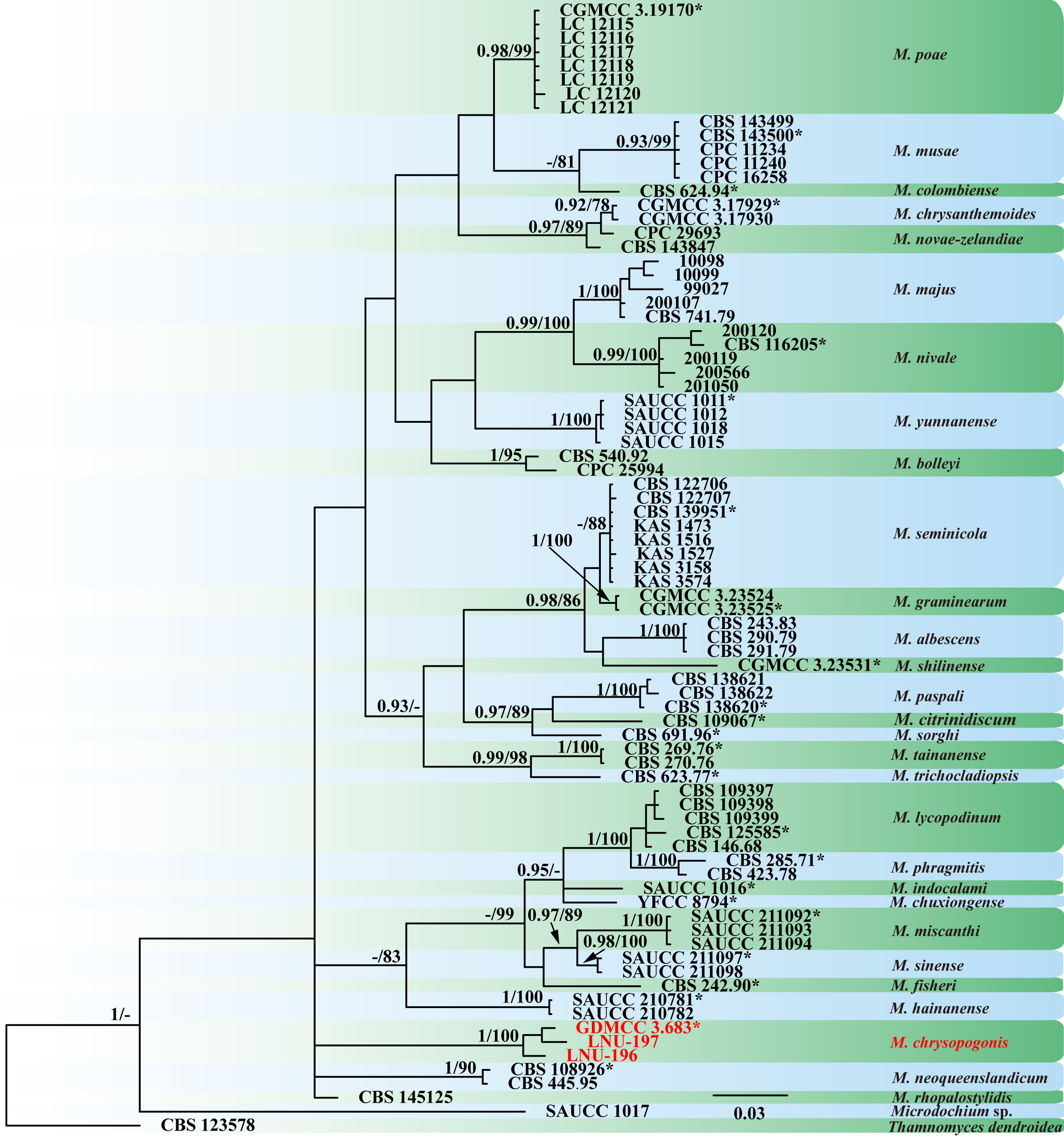

Supplement: Supplementary material 4 — Phylogenetic tree inferred from a maximum likelihood analysis based on a combined alignment of tub2 sequences of 80 isolates of the Microdochium sp. [file mycokeys-100-205-s004.tif]
